# Knowledge, Attitude and Practice of Carrier Thalassemia Marriage Volunteer in Prevention of Major Thalassemia

**DOI:** 10.5539/gjhs.v7n5p364

**Published:** 2015-06-10

**Authors:** Tahmineh Karimzaei, Qolamreza Masoudi, Mahnaz Shahrakipour, Ali Navidiyan, Abd Al-Qaffar Jamalzae, Ahmad Zoraqi Bamri

**Affiliations:** 1 Health promotion Research Center, Zahedan University of Medical Sciences, Zahedan, IR Iran; 2 Faculty of Zahedan University of Nursing & Midwifery, Zahedan, IR Iran; 3 Iranshahr University of Medical Sciences, Iranshahr, Iran

**Keywords:** thalassemia, Health Belief Model (HBM), marrying partners, prevention

## Abstract

**Introduction:**

Thalassemia is the most common genetic disorder and rising in the world as a health problem. Due to the criticality of this disease, in our country thalassemia prevention programs are more importance. The aim of this study was investigation of knowledge, attitude and behavior of marrying partners who were thalassemia genetic carriers in prevention of the birth of the children with major thalassemia

**Methods:**

This study was a descriptive-analytic study. Data collection tool was a self-administered questionnaire that included 43 items. The content validity of questionnaire was investigated under the supervision of physicians, experts of health education and promotion. Its reliability was confirmed by Cronbach’s Alpha test. The subjects in the study consisted of 100 marrying partners who were genetic carriers of thalassemia who referred to Premarital Counseling Center in Iranshahr City. Iranshahr is a a large city of Sistan and Balouchestan Province that located in southeast of Iran. The subjects were selected by convenience non-probability sampling method. Data analyzed using descriptive and analytic statistical tests in SPSS 16.00 and level of significance considered on α < 0.05.

**Findings:**

The average age of men and women that participated in this study was 21.92 and 24 years respectively. 88% of the partners had familial relationships. The educational level of most of the men (34%) was diploma and of women (44%) was pre-diploma. The research findings showed that 7% and 62% of the subjects had poor and mediocre levels of knowledge respectively. Also results showed that only 13% of them had a satisfactory behavior and educational status had a positive correlation with knowledge, behavior, perceived susceptibility and perceived severity (P<0.05). As well there was a significant statistical relationship between gender and familial relationship, and the perceived barriers of participants. (p=0.01).

The survey viewpoint of participants showed that they believed knowledge increasing (40%), genetic counseling (33%) and premarital screening (27%) were the most important strategies for prevention of thalassemia.

**Conclusion:**

The perceived barriers were the strongest predictors for preventive behaviors of incidence of major thalassemia in marrying partners, therefor educational interventions should focused on perceived barriers removing in Volunteer marrying partners.

## 1. Introduction

Thalassemia refers to a group of diseases that are caused due to genetic disorder in formation of normal hemoglobin chain. This disease is the most common genetic disease in human being and is considered as a growing health problem in the world (Cunningham, 2008; Khani, Majdi, Azad Marzabadi, Montazeri, Ghorbani, & Ramezani, 2009; Vichinsky, 2005). Thalassemia is a growing global public health problem with an estimated 900,000 births of clinically significant thalassemia disorders expected to occur in the next 20 years (Vichinsky, 2005). Thalassemia is classified into the two alpha and beta groups based on the type of the decreased globin’s chain (Tarbiat Modares University, 2004) and is transmitted as an autosomal recessive. If two partners who are carriers of thalassemia get married, based on the probability, their children will have severe thalassemia (25%), will be healthy (25%) and be carriers of thalassemia (50%)(Robert et al., 2007).

Almost all different types of thalassemia can be seen in all parts of the world but the disease is more common in the regions of the Mediterranean, the Equador, Africa and Asia. The Mediterranean shore (Italy and Greece), the Arabian peninsula, Turkey, Iran (Debozorgian et al., 2015). The genetic prevalence of thalassemia in these regions is between 2.5 and 15%. Worldwide, Hb E-β-thalassemia is one of the most frequent hemoglobinopathies. The incidence of Hb E approaches 60% of the populations in many regions of Southeast Asia. In coastal regions of North America, its prevalence is rapidly growing (Vichinsky, 2005). The alpha- thalassaemia type is mostly seen in Southeast Asia (India) and also in west coast of Africa (Monica et al., 2015). Based on the studies conducted in the east desert regions of Saudi Arabia, more than 50% of people suffer from a kind of alpha-thalassemia syndrome. Also, generally, 3% of the world population are the genetic carrier of thalassaemia but this rate is higher in some regions such as Italy and Greece (Haghshenas & Zamani, 2008; Vichinsky, MacKlin, Waye, Lorey, & Olivieri, 2005).

In Iran, thalassemia, especially beta-thalassemia, is a high prevalence disease especially in northern of Iran. Also the number of thalassemia patients in some areas such as Fars and Sistan and Baluchestan are remarkable (Ministry of Health and Medical Education of I.R IRAN, 1997-2001). Carriers are easily detected by routine hematological methods and can be forewarned of their reproductive risk. Carriers of structural variants have 30–50% of the variant hemoglobin in their red cells: thalassemia carriers have small red blood cells and sometimes mild anaemia (Weatherall & Clegg, 2001) and β thalassemia carriers also have over 3.5% of Hb A2. The resemblance between thalassaemia and iron deficiency can confuse the diagnosis of either disorder (Wonke, 2007).

In Iran, more than 25000 people are suffering of major thalassemia (Ghotbi & Tsukatani, 2005; Abdolsamadi, Torkzaban, & Hosseini, 2008) and its prevalence are from 3 to 100 people per 100000 people that are distributed in different provinces of Iran. Sistan and Baluchestan Province is located in southern of Iran with a population more than over 2.7 million persons have 2300 patients that are suffering from major thalassemia (85 people per 100000 people) (Abolghasemi et al., 2007).

Thalassemia has not a definite treatment or its treatment is applicable with some limitations. Today maintenance treatment, aimed at patient survived, is conducted through blood transfusion. The annual needs of these patients are met by 50600 units of blood donation. In Sistan and Baluchestan Province, annually about 72000 units of blood donation and 70% of these blood donations are used for thalassemia patients. While in the whole country, about 25% of the blood donations are used for thalassemia patients (Abdolsamadi, Torkzaban, & Hosseini, 2008).

The heart and endocrine glands are harmed most by iron increasing in blood system and in many cases, although the patients tolerate anemia, they die due to heart and glandular failure (Zendehbad, Azadeh Sadat, Mir Behbahani, & Narges Beigom, 2009). Based on the studies conducted, from among 1069 patients with major beta-thalassemia, 16 people (1.5%) die of severe complications of heart, 19 people also have severe heart problems and must be treating by special heart treatments and 106 patients also (9.9%) have average heart injury (Ansari, Vosoogh, Razavi, & Nojoomi, 2003). Thalassemia is not only a health problem but also a socioeconomic problem in many countries (Miri Moghaddam, Narooeinejad, & Eshghi, 2005). Travel and medicine cost (63.8%) were the greatest economic problem of families (Arbabisarjou, Karimzaei, & Jamalzaei, 2015).

According to the rather high prevalence of the disease, at present thalassemia prevention programs are of considerable importance and in our country the prevention programs are specifically based on screening and premarital counseling (Seyam & Assemi, 2010); and national efforts for prevention of thalassemia in the country have started since the last two decades (Kosaryan, Vahidshahi, R. Seyami, Nazari, Karami, & Ehteshami, 2009). Many interventions have been made to increase awareness and decrease the prevalence of the disease in Iran while in Sistan and Baluchestan Province, due to different reasons such as tribal and cultural differences and false beliefs, these interventions have not been successful (Rezaei Kikha, 2012). The success of prevention programmes for control of thalassemia in countries like Cyprus, Italy and Greece have shown the education, teaching to parents and screening are the most important part of these programmes (Angastiniotis et al., 1995; Cao et al., 2002; Loukopoulos, 2011).

The present study is a theory-based educational intervention which is carried out based on framework of HBM, which is one of the patterns of behavioral study in health education and was designed in 1950s by social psychologists (Glanz, Rimer, & Viswanath, 2008). Based on health belief model assumes, the behavior of every person is influenced by the person’s perceptions of different aspects of behavior and the change of these perceptions can lead to behavioral changes. This model suggests some structures for behavioral changes, which include: The perceived susceptibility (i.e. to what extent a person is predisposed to certain diseases), the perceived severity (the person’s beliefs regarding the severity and seriousness of the disease), perceived benefits (the person’s perception of the profits and interests resulting from adopting preventive behaviors), perceived barriers (the person’s perception of the barriers and problems in performing any health behavior and practice), guide for practice (the stimuli that accelerate the decision-making process and create the need to perform a behavior) and self-efficiency (the person’s firm belief in his/her ability in the successful performance of a behavior) (Glanz, Rimer, & Viswanath, 2008).

The study of knowledge and attitude of the marrying partners who are genetic carriers of thalassemia is helpful to determine their thalassemia preventive behaviors is a proper procedure for planning of effective educational interventions in the future, this study was conducted with the goal of investigation of awareness, attitude and behavior of the marrying partners who are genetic carriers of thalassemia, based on the HBM of Iranshahr City regarding thalassemia.

## 2. Methods

This study was a descriptive-analytic study which was performed with the goal of determination of the level of knowledge, attitude and behaviors of the marrying partners who are genetic carriers. They selected via convenience non-probability sampling method. Participants diagnosed through thalassemia blood and genetic diagnostic tests. Marrying partners refer to Premarital Counseling Center of Iranshahr City

The data collecting tool was a mutual self-administered questionnaire which included demographic status (5 items) and health belief model questions included knowledge questions (11 items), behavior (7 items), perceived susceptibility, perceived severity, perceived benefits and perceived barriers each one had five questions and for self-efficiency and keys to action using two questions respectively.

The content validity of questionnaire was investigated under the supervision of physicians and health education and promotion specialists and its reliability was confirmed by Cronbach’s Alpha test. Based on Alpha test, all questions’ reliability was higher than 76% and questions’ validity was higher than 80%. Also, for the convenience of the items in the questionnaire, it was given to 15 people of target group and their views were collected and the necessary changes were made.

For scoring responses of knowledge questions for correct responses, incorrect responses and “I don’t know” scoring was as following: 2 points for “correct” answers, 0 point for “incorrect” answers, and 1 point for “I don’t know” answers. Knowledge response scores were divided to 3 groups: poor (0-7), average (8-15) and Satisfactory level (16-22). Behavior questions were scored in 1 point score for no and 2 point for yes responses. Considering the totally scores of behavior responses all participants in this study were divided to 3 groups as following: Satisfactory level (13-14), mediocre (10-11) and poor (7-8).

The responses of questions of health belief model constructs included perceived susceptibility, perceived severity, perceived benefit, perceived barriers and self-efficiency were designed in three levels contained correct, incorrect and I don’t know that were responded fallowed as: 3, 2 and 1 point respectively. The responses of participants to keys to action questions were calculated by percentage method for each question. After collection, the data was analyzed by SPSS 21 software using descriptive analytic methods. In this study the level of significance considered as P<0.05.

## 3. Findings

The research finding showed that the average ages of the participants were 21.92 and 24 years respectively. 88% of the partners had a familial relationship and also 12% of them were illiterate. The findings indicated that only 20% of the subjects had a satisfactory level of knowledge regarding to thalassemia prevention behaviors. Furthermore, the analysis of the findings showed that in the areas of perceived susceptibility, the perceived severity and the perceived benefits, only 20, 40 and 64% of the people had a satisfactory score respectively (Table 1). Also, in the area of perceived barriers, 58% of the people had high perceived barriers. In the area of perceived self-efficiency, 69% had a high level of self-efficiency and investigation of thalassemia prevention behaviors showed that only 26% of the people had a satisfactory level of behavior (Table 1).

**Table 1:** Descriptive Statistics of knowledge, attitude and all of health belief model structures in marital partners who are thalassemia carriers

Variable	Score domain	Average	Standard deviation	Min	Max	Satisfactory Frequency	Average Frequency	Poor
knowledge	0-22	11.48	4.19	2	21	20	60	20
Behavior	7-21	9.92	1.97	7	14	26	43	31
Perceived susceptibility	5-15	9.77	2.16	5	15	20	55	25
Perceived severity	5-15	11.22	2.24	5	15	40	53	7
Perceived benefits	5-15	12.22	2.07	5	15	64	33	3
Perceived barriers	5-15	7.74	2.35	5	15	5	27	68
Self-efficiency	2-6	5.46	0.93	2	6	69	27	4

The most common key to action for prevention behaviors was the radio and TV (45%) and the least source was the family members (4%). The findings showed that via viewpoint of participants the most important strategy for prevention of major thalassemia must be increasing the awareness (40%), genetic counseling (33%) and premarital thalassemia screening (27%).

The results showed that there was a positive and significant correlation (r=0.274, p=0.006) between behavior and perceived barriers. Also, there was a positive and significant correlation between education and knowledge (r=0.325, p=0.001), perceived susceptibility (r=0.373, p<0.05) and perceived severity (r=0.229, p=0.022). (Table 2)

**Table 2:** correlation among HBM components in marrying partners

	Awareness	Perceived susceptibility	Perceived severity	Perceived benefits	Perceived barriers	Self-efficiency	Behavior
Awareness							
Perceived susceptibility	0.499 0.0001						
Perceived severity	0.359 0.0001	0.616 0.0001					
Perceived benefits	0.327 0.001	0.212 0.34	0.462 0.0001				
Perceived barriers	0.131 0.194	-0.210 0.036	0.012 0.906	0.269 0.007			
Self-efficiency	0.174 0.083	-0.032 0.752	0.244 0.014	0.192 0.56	0.311 0.002		
Behavior	0.082 0.418	-0.035 0.727	0.092 0.360	0.159 0.114	0.274 0.006	0.143 0.157	

## 3. Discussion

The results showed that the mean scores of knowledge, perceived susceptibility and behavior of participants regarding to thalassemia preventive behaviors were low and only 20% of them had a satisfactory knowledge. Also, the results of Pearson’s correlation test indicated that there is a positive statistical relationship between knowledge and perceived susceptibility, perceived severity and perceived benefits, and there is a negative relationship between knowledge and perceived barriers. Thus, based on the assumptions of the HBM and many studies conducted, these perceptions are the predictors and mediators of the behavioral changes. Based on these results, it can be concluded that by conducting educational interventions, the level of awareness of partners who are genetic carriers of thalassemia can be increased and increase of awareness can increase the perceptions of the perceived susceptibility, perceived severity and perceived benefits of people and also decrease the perceived barriers, and these changes can increase the thalassemia prevention health behaviors in partners and consequently the prevalence of major thalassemia will decrease (Sedqiani & Farshidifar, 2001).

The present study showed that the Perceived barrier was the most important predictor of preventive behaviors but a study in Montana men regarding laboratory diagnosis examination of Colorectal cancer (Cyr et al., 2010) showed that the perceived benefits was the strongest predictive factor.

In the study by Asare entitled “prediction of more healthy sexual behaviors among African emigrants”, there was a significant relationship between the perceived benefits (Asare, 2011) and perceived susceptibility, and sexual behaviors.

Finding of this study showed that there was a positive and significant correlation between the perceived severity (seriousness of the disease) and perceived benefits or efficacy of adopting preventive behavior and there was a negative and significant correlation between perceived susceptibility and the person’s perception of the barriers and problems in performance of health behavior. This result is supported by some article such as results of a study that was conducted regarding measuring HBM components in recognition of strongest predictors of adopting behaviors showed that the perceived barriers and knowledge were the strongest predictors of preventive behaviors of cervical cancer (Namdar et al., 2012).

Thus, it can be concluded that planning based on health belief model by focus on perceived barriers and knowledge increasing can lead to improvement of health behaviors to prevention of incidence of major thalassemia in marital partners. The results of a study in Malaysia showed that a there is a low level of knowledge about thalassemia and highlighted the urgency of implementing effective public education programmes. Also, the results cleared the necessity of a holistic approach where public education and promotional activities should be accorded to local cultural and religious beliefs (Li Ping et al., 2011).

The present study showed that 88% of participants (marriage partner volunteer) had a first degree familial relationship. It is determined that consanguineous marriages, due to genetic similarities the prevalence rate of genetic diseases such as major thalassemia were increased, therefore theoretical-based educational interventions for behavioral change or health behavior improvement are highly necessary (Rezaei Kikha, 2012) (Jafari et al., 2004; Qazanfari et al., 2010).

Cultural and racial factors and knowledge shortage regarding adverse results of consanguineous marriages are the most important factors that have an effective role in high prevalence of consanguineous marriages in this region.

Because of these results the most important procedures for prevention of major thalassemia in marital partner volunteer are includes awareness increasing regarding high risk results of consanguineous marriages especially to born major thalassemia newborn and importance of accomplishment of every prophylactic behavior such as Using contraception method, Prenatal genetic diagnostic testing, and perform an abortion considering the official and formal method of Islamic republic of Iran. Considering the beliefs and cultural differences and use of proper media are very important for every success in reduction of major thalassemia incidence.
